# Influence of co-registration on lesion characterization in diffusion-weighted breast MRI

**DOI:** 10.1007/s10334-026-01324-z

**Published:** 2026-01-31

**Authors:** Luise Brock, Andrzej Liebert, Hannes Schreiter, Dominika Skwierawska, Chris Ehring, Jessica Eberle, Shirin Heidarikahkesh, Frederik Bernd Laun, Michael Uder, Lorenz Kapsner, Judith Lach, Evelyn Wenkel, Sabine Ohlmeyer, Dominique Hadler, Florian Knoll, Sebastian Bickelhaupt

**Affiliations:** 1 Institute of Radiology, Universitätsklinikum Erlangen, Friedrich-Alexander-Universität Erlangen-Nürnberg (FAU), Maximiliansplatz 3, 91054 Erlangen, Germany; 2 Department Artificial Intelligence in Biomedical Engineering, Friedrich-Alexander-Universität Erlangen-Nürnberg, Erlangen, Germany; 3 Institute of Computer Science, Polish Academy of Science, Warsaw, Poland; 4 Medical Informatics, Friedrich-Alexander-Universität Erlangen-Nürnberg, Erlangen, Germany; 5 Diagnostische Radiologie, Radiologie München, Munich, Germany; 6 Center for Advanced Imaging Innovation and Research (CAI2R), Department of Radiology, New York University Grossman School of Medicine, New York, NY USA

**Keywords:** Diffusion magnetic resonance imaging, Breast cancer, Comparative study, Medical image processing

## Abstract

**Objective:**

To evaluate if co-registering Diffusion-Weighted Imaging (DWI) before generating Apparent Diffusion Coefficient (ADC) maps can improve differentiating benign and malignant breast lesions in MRI based on the A6702 thresholds.

**Materials and methods:**

This IRB-approved study involved an in-house dataset and the publicly available ACRIN-6698 dataset, both including multi *b*-value DWI. In phase one, 16 ANTs library-based co-registration methods were evaluated on a subset of *n* = 138 cases from our in-house cohort. The quantitative assessment included mean ADC values of manually segmented lesions (diagnostic metrics using individual and A6702-defined thresholds) and coefficient of Variation. In the second phase, the best-performing methods were tested for generalizability on an unseen set of 40 cases (20 from in-house and 20 from external dataset). Three blinded readers segmented lesions on co-registered and non-co-registered ADC maps. Agreement and consistency were evaluated via Bland–Altman, segmentation distance, and intraclass correlation coefficient.

**Results:**

Rigid co-registration using DWI at *b* = 750 s/mm^2^ as reference (b750-Rigid) improved accuracy of both optimal/conservative A6702 trial thresholds with sensitivity/specificity increasing from 93%/10% to 97%/30% and 100%/30% respectively. Mean ADC values were not significantly different after co-registration (*p* > 0.05).

**Discussion:**

Co-registration of DWI images before ADC map generation, particularly using b750-Rigid registration, seems promising for improving lesion classification in breast MRI. Further validation is warranted.

**Supplementary Information:**

The online version contains supplementary material available at 10.1007/s10334-026-01324-z.

## Introduction

In breast cancer diagnostics, breast MRI plays an increasing role for screening high-risk patients, preoperative staging of breast cancer, and post-therapeutic monitoring [1]. Breast MRI is commonly based on a multiparametric imaging protocol, which includes various unenhanced and dynamic contrast-enhanced (DCE) sequences. However, its specificity – that is its ability to correctly classify benign lesions– can be limited [2].

Diffusion-weighted imaging (DWI) has been suggested to potentially provide complementary tissue information in lesions, further improving the lesion characterization without the need for contrast agents [3–6].

DWI indirectly assesses tissue microstructure by analyzing the Brownian motion of water. By calculating the apparent diffusion coefficient (ADC) map from different diffusion-weighted images, the size of the diffusive motion can be quantified. The ADC is a valuable marker to differentiate between benign and malignant lesions and may also help predict treatment response in breast cancer patients undergoing neoadjuvant chemotherapy [4, 7].

Despite its potential, DWI faces technical challenges such as distortion artifacts, and fat saturation issues that can result in motion artifacts. In addition, acquisition time increases with higher spatial resolution or multiple *b*-values, which can further enhance the risk of motion artifacts [8–10]. In this context, the term “motion artifact” also includes distortion-related effects, such as susceptibility or eddy-current distortions, that produce misalignment similar in appearance to motion. These issues can affect the alignment of images acquired with different diffusion-weightings (*b*-values) and therefore affect the accuracy of ADC maps, thereby compromising the diagnostic performance.

The question of this comparative study is whether co-registration before the creation of ADC maps can influence the ADC map quality in terms of the diagnostic performance in breast DWI by reducing misalignment caused by motion, distortion and other sources. Building on prior research presented at WBIR 2024 [11], this study aims to evaluate different co-registration methods from the Advanced Normalization Tools (ANTs) [12] library to improve ADC maps. The study consists of two parts:

- Evaluation on in-house dataset: This part focuses on identifying the best co-registration method by testing 16 different co-registration methods on an in-house dataset and creating a pre-selection of best-performing co-registration methods.
- Evaluation of impact on human reader lesion segmentation on multiple datasets from in-house and from external datasets: This part extends the evaluation of the best performing co-registration methods to two new datasets to assess the impact on human reader lesion segmentation and generalizability of the selected methods of part one.

## Materials and methods

### Datasets

**Fig. 1:**
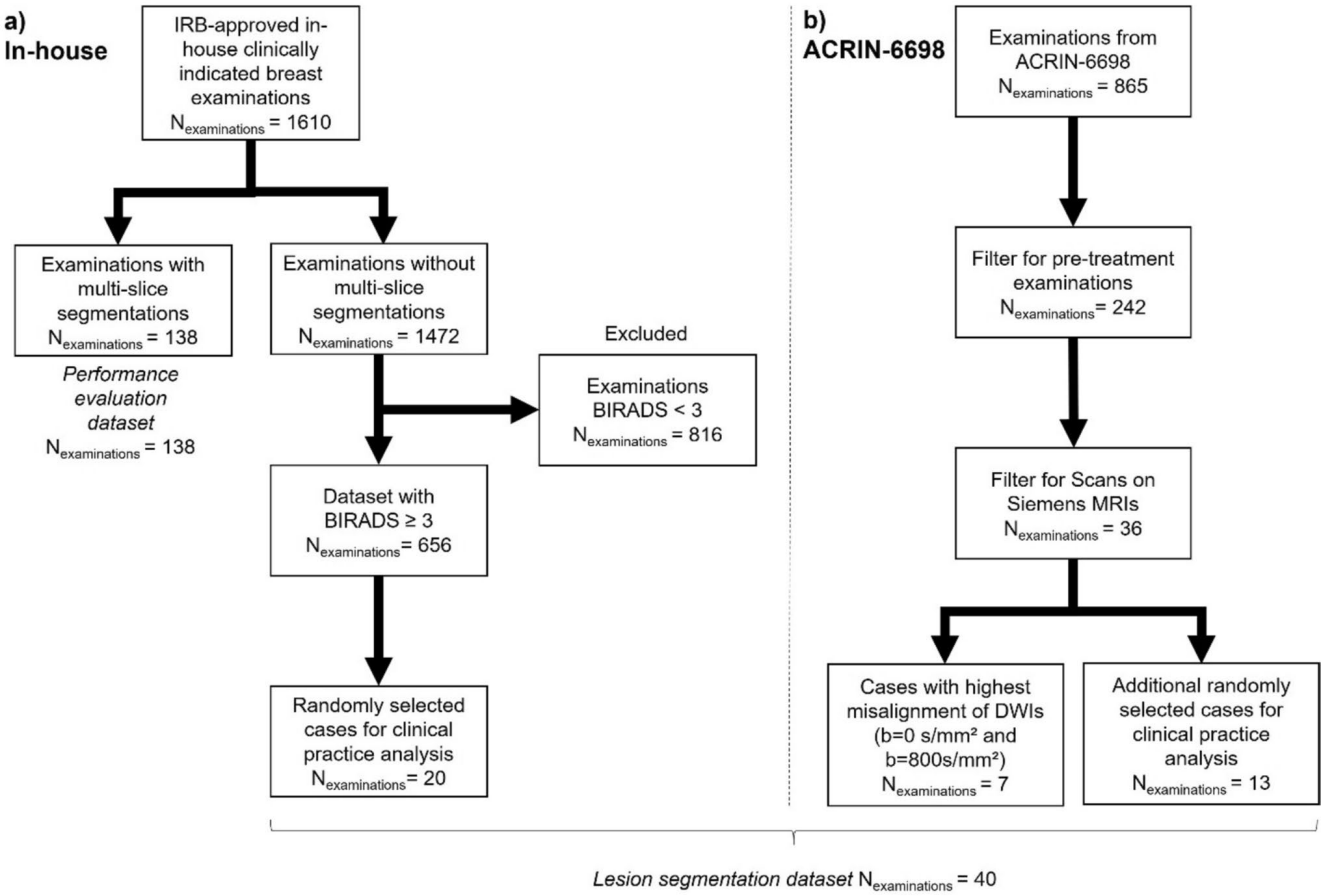
Flowchart illustrating the selection and exclusion criteria for the in-house (**a**) and ACRIN-6698 (**b**) datasets. From an initial 1610 in-house breast MRI examinations, 138 cases with volumetric segmentations were selected for the performance evaluation dataset, while the remaining 1472 cases formed the basis of the evaluation of the impact on human reader lesion segmentation subset. After excluding cases with BI-RADS < 3, a random selection of 20 cases from the remaining 656 was included in the final analysis. For the ACRIN-6698 dataset, 865 cases were initially available. After filtering for pre-treatment examinations and limiting to Siemens-manufactured data, 36 cases remained. From these, the seven cases with the highest DWI misalignment and 13 additional random cases were selected, yielding a total of 20 cases. The final lesion segmentation dataset consisted of 40 cases from both datasets combined

This IRB-approved retrospective study utilized two distinct datasets: An in-house dataset from the University Hospital Erlangen, Germany (called the in-house dataset) and a subset of the ACRIN-6698 dataset (called ACRIN-6698) (Fig. 1).

The in-house dataset contains clinically indicated diagnostic routine breast MRI examinations performed at the Institute of Radiology of the University Hospital Erlangen, excluding patients with breast implants, comprising n = 1610 MRI examinations performed between 2015 and 2020, with a mean patient age of 50 years. (± 13 years). MRI scans were performed in prone position on both 1.5 Tesla and 3 Tesla systems (Magnetom Aera 1.5T and Skyra Fit 3T; Siemens Healthineers, Erlangen, Germany) using a dedicated 18-channel breast coil (Siemens Healthineers). This dataset included a multiparametric MRI protocol with pre- and postcontrast T1-weighted sequences (T1w), T2-weighted sequences, and multi-*b*-value DWI sequences, acquired with a single-shot echo-planar imaging (EPI) sequence, in axial orientation with *b*-values of 50 (b50), 750 (b750), and 1500 (b1500) s/mm^2^ (detailed acquisition parameters for DWI are provided in Supplemental Table 1). For DWI vendor specific distortion correction steps have been applied during image reconstruction. Histopathology results served as the ground truth and were retrieved from the hospital information system.

Apart from the prior work on co-registration of ADC, published as part of the 11th International Workshop, WBIR 2024 conference proceedings [11], the data were previously included in different studies on virtual breast image generation [13–17], detection and forecasting of artifacts [10, 18–20], incidental finding [21] and skin pathology imaging [22].

The second dataset consisted of the ACRIN-6698 dataset, which included *n* = 865 cases with invasive breast cancer [23]. Details on the dataset, including the underlying study and MRI protocols, can be found in the respective reference. For comparability with our in-house data, we filtered it to retain only MRI examinations performed with systems from vendor Siemens Healthineers and which were acquired prior to adjuvant chemotherapy. This resulted in *n* = 36 breast MRI examinations. The MRI systems used included 1.5 Tesla and 3 Tesla Siemens models (Magnetom Avanto 1.5T, TrioTim 3T, and Prisma fit 3T; Siemens Healthineers, Erlangen, Germany) with sequences including pre-contrast T1w, post-contrast T1 subtraction series, and DWI, acquired with a single-shot EPI sequence, with *b*-values of 0 (b0), 100 (b100), 600 (b600), and 800 (b800) s/mm^2^.

All data were transferred to dedicated research workstations. The goal of the first part of the study was to evaluate the best-performing co-registration methods, based on a subset of the in-house dataset. The sub-dataset with volumetric segmentations of lesions on b1500 images of *n* = 138 (mean age: 53.2 ± 11.7 years) cases comprised *n* = 68 benign and *n* = 70 malignant lesions. For the *n* = 138 cases, only the largest lesion in each dataset was segmented in 3D on b1500 images using the integrated draw-function of Slicer3D (version 4.11.20210226) [24]. In four of the malignant and one of the benign cases, two lesions were annotated per patient, as there were two major lesions visible. In cases with necrosis, only viable tumor tissue was segmented, and necrotic areas were excluded to ensure consistent representation of intralesional tissue. The segmentations were performed by a medical student (J.E.) with two years of experience in breast DWI under the supervision of a board-certified radiologist (S.B.) with more than 10 years of experience in breast DWI. In the following, this dataset will be called *performance evaluation dataset*.

For the second part of this study, the evaluation of the impact on human reader lesion segmentation analysis, two datasets were used. The first dataset was derived from the in-house dataset, excluding cases used in the performance evaluation, leaving *n* = 1469 available datasets. From these, *n* = 20 (mean age: 49 years) cases were selected randomly, but ensuring an equal number of malignant (*n* = 10) and benign (*n* = 10) cases. The second dataset was derived from the ACRIN-6698 study. For the *n* = 36 Siemens pre-therapy examinations, a visual reading was performed to score the overall image alignment between b0 and b800 DWI sequences, focusing on the alignment of breast tissue and lesions. One reader (L.B.), with one year of experience in breast DWI, evaluated and scored the images using a 5-point Likert scale (1: Very Poor Alignment, 5: Perfect Alignment). All cases received a score of either 4 or 5, indicating at least good alignment. From these, the seven datasets scored as 4 were selected, along with 13 randomly chosen cases, resulting in a total of 20 datasets (mean age: 47 years). The resulting 20 datasets from the ACRIN-6698 study composed of 11 cases from a 1.5T scanner and 9 cases from a 3T scanner. For the evaluation of the impact on human reader lesion segmentation, both datasets were combined, forming a *lesion segmentation dataset* of 40 cases.

### Experimental setup overview

**Fig. 2:**
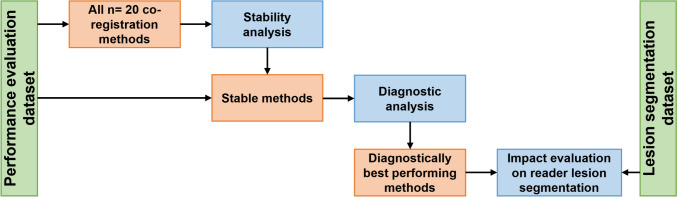
Experimental workflow to assess whether inter-b-value co-registration prior to ADC map creation improves accuracy of ADC and clinical analysis. With the performance evaluation, which is divided into stability analysis and diagnostic analysis, the best-performing methods were selected in comparison to no co-registration based on different comparison criteria and then further validated using the lesion segmentation dataset in an evaluation of the impact on human reader lesion segmentation

Figure 2 shows the experimental workflow of the study performed in order to evaluate the co-registration of the different *b*-values prior to ADC map calculation. First, our initial *n* = 16 different co-registration approaches, which are explained in detail below in Sect. "Co-registration methods", were applied to the DWIs of the *performance evaluation dataset,* and ADC maps were generated from the co-registered and the non-registered DWIs.

A stability analysis of the ADC mean values inside the 2D lesion segmentations using Coefficient of Variation (CoV) and outlier detection was performed to ensure that co-registration does not strongly alter the ADC mean values of lesions and thereby reduce the stability of ADC maps (see Sect. "Stability analysis"). From the 16 initial co-registration methods, we selected the so-called ‘stable’ methods by comparing their performance to that of no co-registration. The exact criteria that were used for selecting the methods after each evaluation step are further described in the following sections. The classification methods that passed the stability analysis as better or equally performing in comparison with *no-coreg* were further assessed in a diagnostic performance analysis, focusing on accuracy, sensitivity, and specificity in distinguishing benign from malignant lesions (see Sect. "Diagnostic performance analysis"). Evaluating the stability analysis, followed by the diagnostic analysis on the 16 co-registration methods, the best-performing methods were selected.

In the second part of the study, ADC maps generated by the best-performing coreg methods were tested against *no-coreg* ADC maps by evaluating the impact on human reader lesion segmentation using the *lesion segmentation dataset*.

### Co-registration methods

The aim of using different co-registration methods is to identify the optimal alignment order and transformation type (rigid or non-rigid) for DWI images, in order to generate the most accurate ADC maps. Accurate ADC maps are essential for differentiating between benign and malignant lesions.

The co-registration approaches are summarized in Table 1. Similar to the WBIR study [11], co-registration methods were based on the ANTs library [12], with the difference of adding b750 as a possible target for co-registration in this study, resulting in *n* = 16 co-registrations using different co-registration types from the ANTs library, with b50, b750, or b1500 images as co-registration targets and applied iteratively or non-iteratively.

**Table 1:** Overview over 16 different co-registration methods with the co-registration type, target and whether it is an iterative or non-iterative method

	Co-registration type	Target for Co-registration	Iterative	Segmentation alignment
**Non-iterative**				
b50-Affine	Affine	b50	No	Yes
b50-Rigid	Rigid	b50	No	Yes
b50-Similarity	Similarity	b50	No	Yes
b50-SyN	SyN	b50	No	Yes
b750-Affine	Affine	b750	No	Yes
b750-Rigid	Rigid	b750	No	Yes
b750-Similarity	Similarity	b750	No	Yes
b750-SyN	SyN	b750	No	Yes
b1500-Affine	Affine	b1500	No	No
b1500-Rigid	Rigid	b1500	No	No
b1500-Similarity	Similarity	b1500	No	No
b1500-SyN	SyN	b1500	No	No
**Iterative**				
b1500-iter-Affine	Affine	b1500	Yes	No
b1500-iter-Rigid	Rigid	b1500	Yes	No
b1500-iter-Similarity	Similarity	b1500	Yes	No
b1500-iter-SyN	SyN	b1500	Yes	No

The last column indicates if segmentations needed to be aligned to the target or not, since they were performed on the target image

Both rigid and non-rigid methods were used, with non-rigid registration addressing potential three-dimensional breast deformation during imaging and distortion artifacts, while rigid registration provided robustness since it preserves the overall shape and volume of structures, avoiding potential unrealistic deformations that can occur with non-rigid transformations.

Across all targets, four types of transformations from the ANTs library were applied: Rigid (rotation and translation only), Similarity (scaling, rotation, and translation), Affine (rigid registration with additional scaling), and SyN (symmetric normalization with affine and deformable transformations)[25]. We focused on the type of transformations for the evaluation and kept the default cost function from the registration function in the ANTs library, which is mutual information.

When b1500 images were the target, co-registration was additionally performed iteratively for each co-registration type, first aligning b750 images to b1500 images, creating an “aligned b750”, then b50 images to the aligned b750 images. For all other targets, co-registration was performed only non-iteratively.

Since we evaluated the co-registration methods based on the ADC mean values inside the lesions, it was necessary to register also the lesion segmentations to the target, when b1500 images were not the target because the segmentations were originally performed on b1500 images (see 2.1 Dataset). In these cases, the b1500 image was first co-registered to the target, and the segmentation was aligned to the target using the same transformation as the b1500 image. In comparison to the WBIR study, the segmentation was then binarized at a 0.3 threshold to maintain consistency with pre-co-registration masks.

A baseline method, *no-coreg*, was included for comparison to evaluate potential improvements. In an additional analysis, we evaluated the influence of masking during co-registration. This analysis has been moved to the supplementary material to keep the main text concise (Supplemental Chapter 1, Supplemental Fig. 2, Supplemental Table 2).

**Table 2:** Patient and lesion characteristics for benign and malignant cases

Characteristics	Benign (n = 68)	Malignant (n = 70)
Age (years)	49.0 ± 11.2	57.0 ± 10.9
Lesion volume (mm^3^)	278 ± 592 107 (49–173)	2,004 ± 4,718 367 (141–1,637)
**BI-RADS scores**		
0	4	1
1	0	0
2	5	1
3	5	0
4	26	15
5	10	39
6	18	14

Age is presented as mean ± standard deviation. Lesion volume is reported both as mean ± standard deviation and as median with interquartile range (IQR) due to the skewed distribution. BI-RADS categories reflect radiological assessment

Recent literature indicates that using only two *b*-values for ADC calculations maintains comparable predictive value and repeatability to using four *b*-values [7, 26]. Hence, our study focused on two-*b*-value based ADC calculations, computed voxel-wise using Eq. (1) where S(b_1_) and S(b_2_) represent the signal intensities at two different *b*-values, one close to 0 and one close to 750 s/mm^2^ [27]. Choosing these specific *b*-values is based on a study of Pereira et al., who found slight improvements using these *b*-values [28]. For in-house data, the closest values to this are b50 and b750, and for ACRIN-6698 data, they are b0 and b800.

1 $$\mathrm{ADC}= \frac{\mathrm{ln}\left(\frac{S\left({b}_{2}\right)}{S\left({b}_{1}\right)}\right)}{{b}_{1}- {b}_{2}}$$

Voxel-wise ADC maps were generated for the entire breast. In the first part, where the best co-registration method is evaluated on the *performance evaluation dataset*, mean lesion ADC was obtained by averaging all voxel values within the volumetric tumor segmentation. In the second part, focused on evaluating the impact on human reader lesion segmentations on the *lesion segmentation dataset*, mean ADC was derived analogously but restricted to the 2D segmentation on the slice with the largest lesion diameter.

### Best co-registration methods analysis

In the first part of the study, the goal was to identify from the *n* = 16 co-registration methods presented in Sect. "Co-registration methods" the best performing co-registration methods. This analysis was done on the performance evaluation dataset and was performed in an iterative way as described in Sect. "Experimental setup overview". First, the stable methods were filtered by the stability analysis. Afterwards, the stable methods were evaluated based on their diagnostic performance analysis. After each analysis, the methods that performed worst in the analysis according to the criteria presented in the sub-sections were discarded from further analysis, resulting at the end in the best performing methods for part two of the study.

#### Stability analysis

We aimed to investigate the stability of the co-registration and ensure that its application does not introduce additional variation in the resulting mean ADC values of the volumetric lesion segmentations. To do this, we analyzed the number of outliers in benign and malignant lesions. Outliers were identified as cases in which the mean value of the ADC in the lesion exceeded 1.5 times the interquartile range (IQR) [29]. Co-registration methods for which the number of outliers exceeded the number of outliers in the non-co-registered data were not considered for further analysis.

Additionally, within-lesion CoVs and Mean were analyzed for benign and malignant lesions. Statistical significance compared to *no-coreg* was assessed using repeated measures ANOVA with Dunnett's post hoc analysis [30], considering p-values < 0.05 as significant. The t-values from the ANOVA with Dunnett’s post hoc test indicate whether the CoV in each method differs significantly from the *no-coreg* condition. Methods with non-significant or lower t-values were considered to have stable or reduced variability. In particular, methods with t-values close to zero or less (indicating no increase or a decrease in CoV compared to no-coreg) were retained, while those with large positive t-values (indicating increased CoV) were filtered out.

#### Diagnostic performance analysis

The diagnostic analysis focused on the clinical impact of co-registration on distinguishing benign from malignant lesions. Performance metrics—including area under the receiver operating characteristics curve (AUROC), sensitivity, specificity, and accuracy—were calculated for each method using receiver operating characteristic (ROC) curve analysis and on individually calculated optimal cutoff based on the Youden index (called individual threshold in the results section) [31]. Additionally, the previously published optimal ADC cutoff (1.53 × 10⁻^3^ mm^2^/s) and conservative cutoff (1.68 × 10⁻^3^ mm^2^/s) from the A6702 trial were used to calculate diagnostic metrics. The optimal cutoff was originally identified to achieve the best trade-off between sensitivity and biopsy reduction, with values below this threshold indicating a higher likelihood of malignancy. An alternate conservative cutoff was proposed for fewer false negatives, though at the expense of a smaller reduction in biopsy rates [2, 6]. From here on these two thresholds of the A6702 trial will be referred to as A6702 trial thresholds. Statistical significance was assessed for the cutoff’s predictive accuracy of the co-registration methods compared to the *no-coreg* method using McNemar's test [32].

The three methods with the highest AUROC and the best accuracy were selected for the second part of the study.

### Evaluation of the impact of co-registration on human reader lesion segmentation

In the second part of the study, the previously selected best-performing co-registration methods were further analyzed to evaluate the impact of the co-registration on the segmentations a human reader does on the ADC maps.

The *lesion segmentation dataset,* consisting of *n* = 40 (30 malignant, 10 benign) cases, was used for this evaluation. The ADC maps of the three best-performing co-registration methods that were selected based on the stability analysis (Sect. "Stability analysis") and the diagnostic performance analysis (Sect. "Diagnostic performance analysis")—and *no-coreg* ADC maps—were used for this purpose. To maintain consistency in naming, methods applied to the ACRIN-6698 dataset followed the same *b*-value nomenclature as the in-house dataset. While ACRIN-6698 data used b0 and b800 instead of b50 and b750, we retained the original naming convention (e.g., b750-*registration type*) to ensure clarity. The resulting ADC maps, along with their respective *b*-value images and contrast enhanced T1-weighted subtraction images at the second time point (T1w-sub2), were randomly assigned into four reading folders. For in-house data, the BI-RADS scores and radiologist reports were added, and for the ACRIN-6698 dataset, it was known to the readers that all lesions were malignant. Three independent readers (D.S., three years of experience in DWI Breast MRI; C.E., two years of experience in DWI Breast MRI; D.H., 15 years of experience in DWI Breast MRI) reviewed these folders, with a two-week interval between readings to minimize recall bias. Blinded to the ADC map generation process, they segmented the main lesion at the slice of its largest diameter using Slicer3D (version 5.6.2) [24]. To find the lesion with the largest diameter, the comments from the radiological report were taken into account for the in-house data and the lesion was first identified in T1w-sub2 and b750/b800 images, to afterwards use the b750/b800 images as an overlay over the ADC map, and to then segment on the ADC map using the integrated draw-function of Slicer3D. With regard to intralesional heterogeneity, readers were explicitly instructed to exclude necrotic components from their segmentations. In cases of differing segmentation targets chosen by the readers, the most experienced reader (D.H.) determined the correct lesion, and segmentations were adapted to this lesion. The analysis assessed segmentation accuracy and ADC values to evaluate co-registration performance.

#### ADC map segmentation evaluation

Segmentation accuracy on the ADC maps was evaluated using several metrics. Bland–Altman analysis compared 2D lesion segmentation areas between non-co-registered and co-registered ADC maps, plotting the mean area difference against the overall mean area across readers. Spatial consistency was evaluated by calculating the mean distance between segmentations from *no-coreg* and co-registered ADCs, differences in lesion positioning were evaluated using the Friedman test, assuming a *p* value < 0.05 to be significant [33].

Reader agreement was evaluated to determine whether image registration influenced the consistency of lesion segmentations across readers. Agreement was quantified using the Intraclass Correlation Coefficient (ICC), applied separately to distances between reader segmentations and lesion area measurements. ICC3k was applied, as it assesses absolute agreement in a two-way mixed-effects model, appropriate when the set of raters is fixed and the focus is on the consistency of their ratings across all subjects [34]. While overlap-based measures such as the Dice coefficient are commonly used for segmentation evaluation, we did not apply Dice in this context because co-registration can introduce slight in-plane or through-plane shifts of the lesion. These small spatial displacements can substantially reduce Dice scores despite segmentations being clinically equivalent, potentially resulting in misleading conclusions. Therefore, our evaluation focuses on lesion segmentation position and size, as these metrics more directly capture the impact of co-registration on lesion extent and displacement.

Additionally, pairwise comparisons of the distances between lesion segmentations of different methods were performed to evaluate the impact of registration on reader agreement. For each reader pair, the Wilcoxon signed-rank test was applied to compare the distances between the reader segmentations of the reference method (*no-coreg*) against each registration method, with a *p* value < 0.05 meaning statistically significant. The same was done for the relation of the segmentation areas between the reader pairs for the different methods.

#### Quantitative ADC values analysis

Firstly, we tested for statistical significance between the ADC mean values in the 2D lesion segmentation of no-coreg and the co-registration methods for benign and malignant lesions separately using repeated measures ANOVA with Dunnett's post hoc analysis, considering *p* values < 0.05 as significant. Afterwards, for each method, the diagnostic performance was analyzed, individually for each reader and method, using the AUROC and a DeLong’s test for statistical significance evaluation. Additionally, AUROC was created for the averaged ADC means inside the 2D lesion segmentations across the three readers for each method. For the averaged ADC means, sensitivity, specificity, and accuracy were evaluated at three distinct cutoffs similar to the diagnostic performance evaluation from above: the optimal individual cutoff determined for each method, the A6702 trial optimal and conservative cutoff. The statistical significance of the differences in the predictive accuracy between the methods and *no-coreg* at the different cutoffs was calculated using McNemar’s test, with *p* < 0.05 meaning statistical significance.

## Results

### Best-performing co-registration method evaluation

This part of the results focuses on getting the best performing co-registration methods based on technical and diagnostic evaluations on the *performance evaluation dataset* with *n* = 138 datasets, comprising 68 benign and 70 malignant cases with volumetric segmentations. The lesion demographics are shown in Table 2, including mean age, lesion size, and BI-RADS scores of the two lesion types. Lesion volume varied widely in both groups, with a notably skewed distribution. The mean lesion volume was 279 ± 592 mm^3^ in the benign group and 2004 ± 4717 mm^3^ in the malignant group. Median volumes were substantially lower (107 mm^3^ and 367 mm^3^, respectively), reflecting the presence of outliers in both groups.

#### Stability analysis

On the initial 16 co-registration methods, the outlier analysis showed an increase in the number of outliers for *b50-Affine, b50-Similarity*, *b50-SyN, b750-SyN, b1500-SyN*, and *b1500-iter-SyN* with either more benign or more malignant outliers, see Table 3. No-coreg showed only one outlier for each lesion type.

**Table 3:** Results of the stability analysis presenting outliers for malignant and benign ADC mean analysis using the IQR method defining outliers as cases in which the mean value of the ADC in the lesion exceeded 1.5 times the interquartile range

	Stability analysis	
	Benign outliers	Malignant outliers
no-coreg	1	1
b50-Affine	1	**2**
b50-Rigid	1	0
b50-Similarity	0	**4**
b50-SyN	**5**	1
b750-Affine	1	1
b750-Rigid	1	0
b750-Similarity	1	1
b750-SyN	**3**	**2**
b1500-Affine	1	0
b1500-Rigid	0	0
b1500-Similarity	0	0
b1500-SyN	**5**	0
b1500-iter-Affine	0	0
b1500-iter-Rigid	1	0
b1500-iter-Similarity	1	1
b1500-iter-SyN	**2**	**3**

Highlighted in red the non-stable co-registration method concerning outliers

**Fig. 3:**
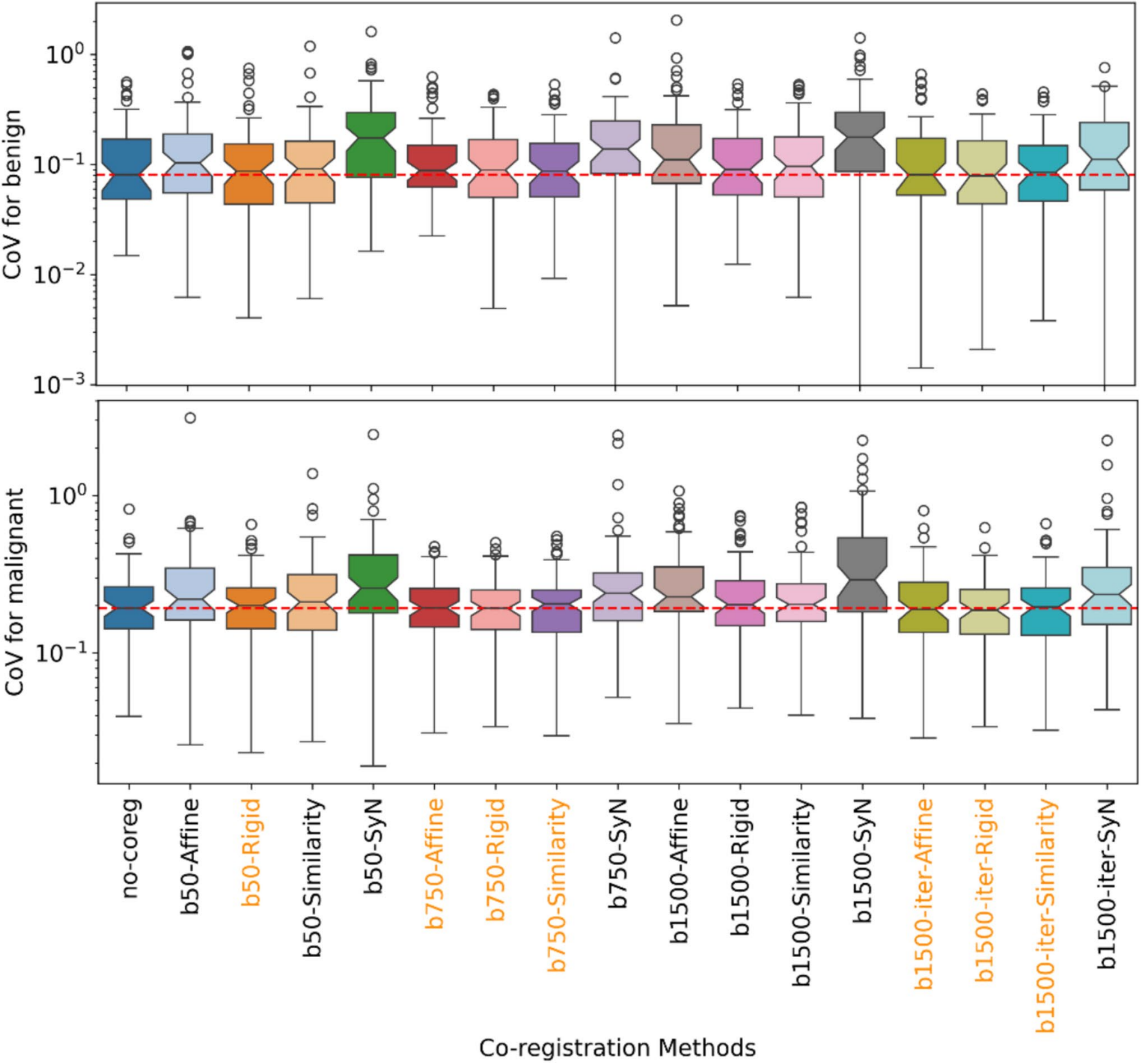
Boxplots for all methods (no-coreg and co-registration methods) showing the CoV for benign and malignant lesions. The red line indicates the median of the no-coreg boxplot and the methods marked in orange are the ones performing similar or better to no-coreg, based on the boxplot and the *p*- and *t*-values from ANOVA with Dunnett’s post hoc test. The scale of the y-axis is logarithmic

Figure 3 shows the analysis of the CoV. Based on the repeated measures ANOVA and the Dunnett post hoc test methods, with t-values below 1 (marked in orange in Fig. 3) were considered comparable to *no-coreg*. The full set of p- and t-values is provided in Supplemental Digital Content (Supplemental Table 3). The analysis of mean showed only significant differences to *no-coreg* for *b50-SyN, b1500-Affine,* and *b1500-SyN*, see Supplemental Content (Supplemental Fig. 1).

After excluding non-stable methods, only *b50-Rigid*, *b750-Affine*, *b750-Rigid*, *b750-Similarity*, *b1500-iter-Affine*, *b1500-iter-Rigid*, and *b1500-iter-Similarity* were used for further analysis.

#### Diagnostic performance analysis

**Fig. 4:**
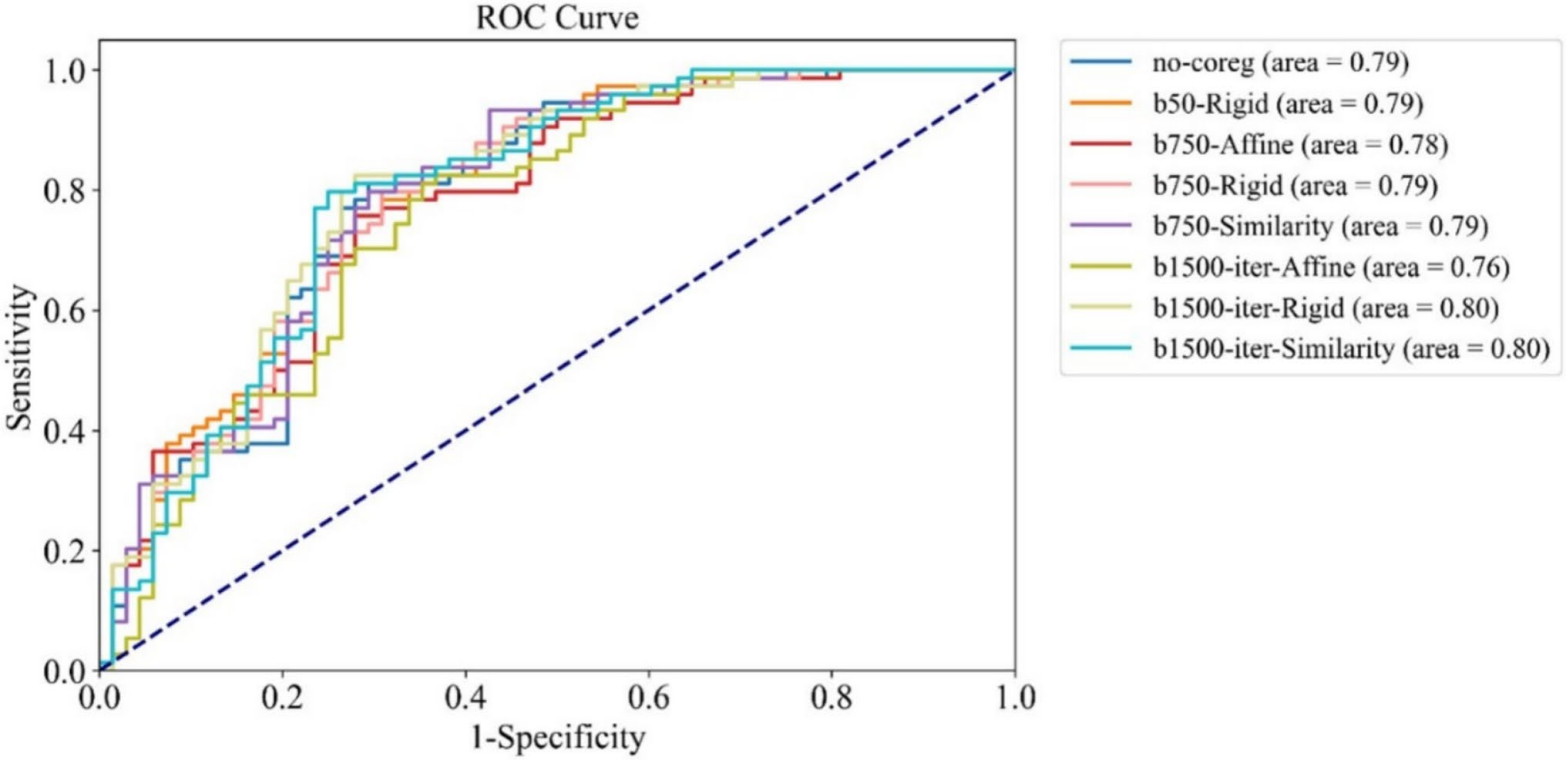
ROC curves based on ADC mean values for differentiating between benign and malignant breast lesions for no-coreg and the technically best performing methods. Legend indicating AUROC for the different methods

The diagnostic evaluation on the *performance evaluation dataset* assessed the potential clinical influence of co-registration on volumetric lesion segmentation by analyzing its effect on the differentiation between benign and malignant lesions. Overall, AUROC values remained stable across the different co-registration methods at 0.79, with differences generally only affecting decimal places within ± 0.01, except for *b1500-iter-Affine* (0.76), indicating reduced diagnostic performance (Fig. 4). Sensitivity, specificity, and accuracy were largely comparable to *no-coreg* (sensitivity: 0.812, specificity: 0.680, accuracy: 0.756), with *b1500-iter-Rigid* and *b1500-iter-Similarity* achieving slightly higher accuracy (0.764).

Using the optimal A6702 trial cutoffs, all methods achieved high sensitivity (≥ 0.957), but had low specificity (0.32–0.38). The conservative threshold further increased sensitivity (≥ 0.986) at the cost of lower specificity (0.18–0.26). *b750-Affine* showed lower accuracy and sensitivity across both thresholds.

No statistical difference in predictive accuracy using McNemar’s test was found compared to no co-registration. The complete diagnostic performance metrics are provided in Table 4.

**Table 4:** Sensitivity, specificity and accuracy for individual thresholds and A6702 trial thresholds for no-coreg and the stable methods, showing for some methods similar results to our previously published analysis [11]

	Sensitivity	Specificity	Accuracy	Threshold (mm^2^/s)
**Individual thresholds**				
no-coreg	0.812	0.680	0.756	1.31 × 10^–3^
b50-Rigid	0.754	0.680	0.723	1.29 × 10^–3^
b750-Affine	0.754	0.700	0.731	1.25 × 10^–3^
b750-Rigid	0.797	0.640	0.731	1.30 × 10^–3^
b750-Similarity	0.797	0.660	0.739	1.27 × 10^–3^
b1500-iter-Affine	0.826	0.640	0.748	1.27 × 10^–3^
b1500-iter-Rigid	0.826	0.680	0.764	1.28 × 10^–3^
b1500-iter-Similarity	0.797	0.720	0.764	1.25 × 10^–3^
**Optimal thresholds according to A6702 trial**				
no-coreg	0.971	0.360	0.714	1.53 × 10^–3^
b50-Rigid	0.957	0.380	0.714	1.53 × 10^–3^
b750-Affine	0.957	0.320	0.689	1.53 × 10^–3^
b750-Rigid	0.971	0.340	0.706	1.53 × 10^–3^
b750-Similarity	0.971	0.320	0.697	1.53 × 10^–3^
b1500-iter-Affine	0.986	0.320	0.706	1.53 × 10^–3^
b1500-iter-Rigid	0.971	0.360	0.714	1.53 × 10^–3^
b1500-iter-Similarity	0.971	0.340	0.705	1.53 × 10^–3^
**Conservative thresholds according to A6702 trial**				
no-coreg	0.986	0.240	0.672	1.68 × 10^–3^
b50-Rigid	0.986	0.260	0.681	1.68 × 10^–3^
b750-Affine	0.986	0.180	0.647	1.68 × 10^–3^
b750-Rigid	0.986	0.220	0.664	1.68 × 10^–3^
b750-Similarity	0.986	0.200	0.665	1.68 × 10^–3^
b1500-iter-Affine	1.0	0.180	0.656	1.68 × 10^–3^
b1500-iter-Rigid	0.986	0.220	0.664	1.68 × 10^–3^
b1500-iter-Similarity	1.0	0.220	0.672	1.68 × 10^–3^

### Evaluation of impact on human reader lesion segmentation

For evaluating the impact on human readers in segmenting lesions, we selected *b50-Rigid*, *b750-Rigid*, and *b750-Similarity*, using *no-coreg* as a comparison method representing the current standard. Ultra-high *b*-value DWI is not included in public datasets, leading to b1500-based co-registration methods being excluded from this analysis. *b750-Affine*, which showed the weakest diagnostic performance, was also removed from further analysis. The evaluation was performed on the *lesion segmentation dataset* with *n* = 40 cases, comprising *n* = 30 malignant and *n* = 10 benign cases.

In Table 5, the lesion characteristics of the in-house half of the *lesion segmentation dataset* is presented. The ACRIN-6698 data consists of *n* = 20 cases, all malignant with no further information about the BI-RADS scores.

**Table 5:** Patient and lesion characteristics for benign and malignant cases. Age is presented as mean ± standard deviation

Characteristics	Benign (n = 10)	Malignant (n = 10)
Age (years)	44.5 ± 16.3	54.3 ± 11.8
**BI-RADS scores**		
0	0	0
1	0	0
2	1	1
3	5	0
4	3	2
5	0	3
6	1	4

BI-RADS categories reflect radiological assessment

**Fig. 5:**
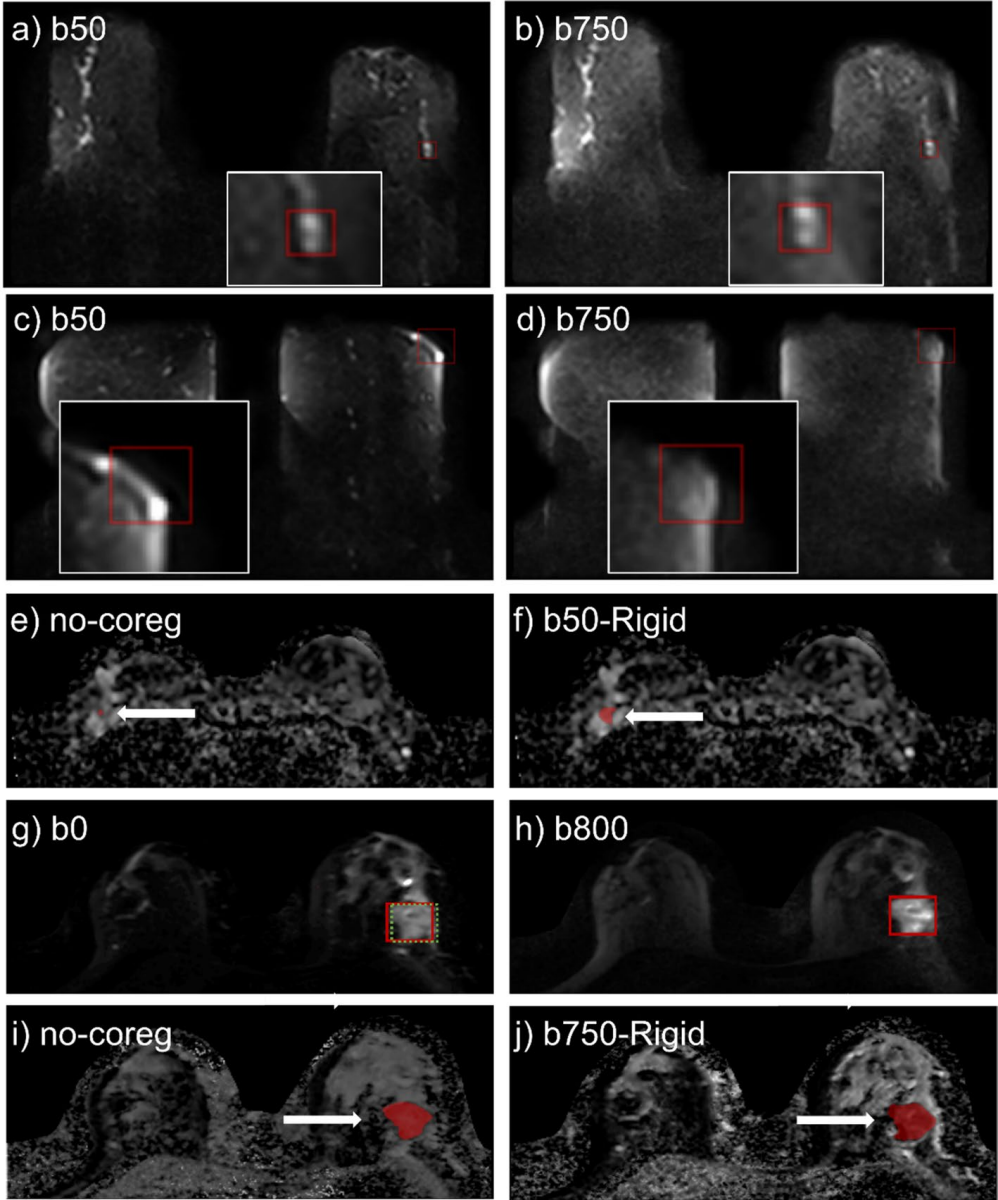
**a** and **b** show an example with slight misalignment of the lesion between the b50 and b750 images, with the lesion shifting a few pixels upwards in the b750 image. **c** and **d** demonstrate a case with misalignment or distortion between b50 and b750 images at the breast borders, not affecting the lesion itself. **e** and **f** show an example, where the same reader created different looking segmentations (red) on the ADC maps of two different methods. **e** ADC Map generated using non-co-registered b-values (no-coreg). **f** ADC Map generated using b50-Rigid co-registration method. **g–j** show an example, where there is a misalignment of the lesions visible in b0 (DWI with b-value of 0 s/mm^2^) and b800 (DWI with b-value of 800 s/mm^2^), yet the resulting lesion segmentations done by the same reader on both ADC maps remain similar. **g** shows the b0 DWI with a dotted green box showing the position of the lesion in b0 and a red box indicating its location in b800. **h** shows the b800 image with a red box indicating the lesion position. **i** ADC map generated using non-co-registered *b*-values (no-coreg). **j** ADC map generated using b750-Rigid co-registration method

To provide a clearer perspective on image misalignment in clinical routine datasets, we have included representative DWI images illustrating lesion misalignment and image distortions between different *b*-value images. Figure 5 shows the general problem of small misalignments and distortions between different *b*-value DWIs. In (A) to (D) two examples of misalignment of the lesion and distortion in the image are shown, to show the need for co-registration. Furthermore, the figure shows examples of segmentations (red) created by the same reader on ADC maps generated using different methods. In (E) and (F), an example dataset is shown where the reader performed different segmentations in size for *no-coreg* and *b50-Rigid* ADC maps. (G)–(J) show a case where even though there is misalignment visible in b0 and b800, the segmentations of the lesions were drawn very similar on ADC maps generated using two different registration processes (no-coreg and *b750-Rigid*).

#### Segmentation evaluation

**Fig. 6:**
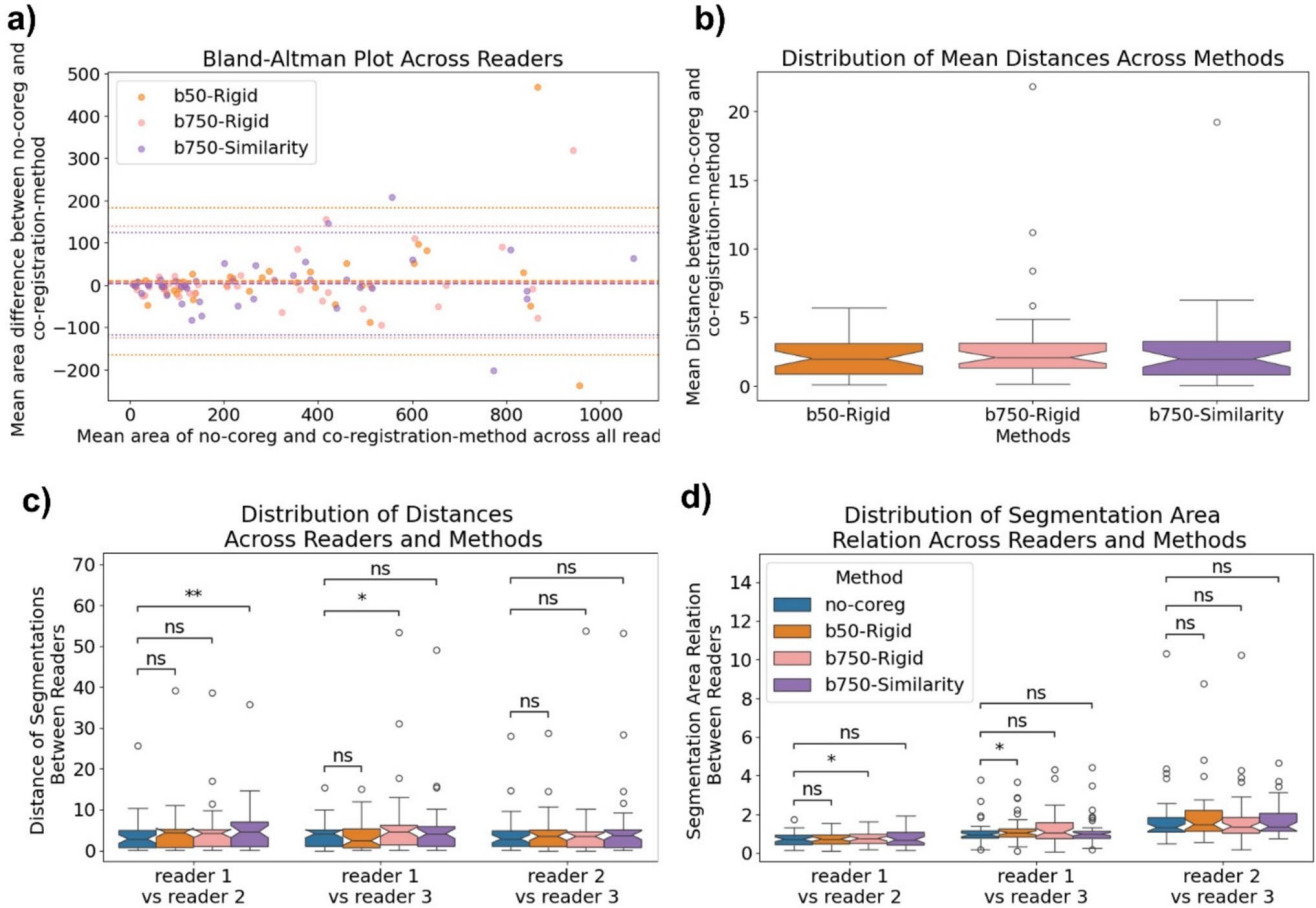
**a** Mean agreement across all readers of lesion segmentations between no-coreg and individual co-registration methods (b50-Rigid, b750-Rigid and b750-Similarity), showing the difference between the mean area across all readers of no-coreg and the mean area across all readers for the individual methods on the y-axis and the mean area of no-coreg and the individual co-registration method across all readers on the x-axis. The dotted lines represent the limits of agreement (1.96 times the standard deviation above and below the mean) for each method and the dashed line represents the mean. Area in mm^2^. **b** The mean distance across all readers between the segmentation using no-coreg ADC maps and the individual methods ADC maps. Distance in mm. **c** Boxplot showing the distance of lesion segmentations between the readers for all methods. Methods legend is shown in Fig. 6d). Statistical significance indicated by ns: non-significant, *p < 0.05, **p < 0.01. Distance in mm. **d** Boxplot showing the area relation of lesion segmentations between the readers for all methods. Statistical significance indicated by ns: non-significant, *p < 0.05, **p < 0.01. Area in mm^2^

Bland–Altman analysis (Fig. 6a) showed small bias for the individual methods compared to *no-coreg*, especially for *b750-Similarity* (*b50-Rigid*: 9.07 mm^2^, **b750-Rigid**: 7.15 mm^2^,* b750-Similarity*: 3.43 mm^2^), with high agreement between methods. *b50-Rigid* had slightly wider limits of agreement (*b50-Rigid*: -165.35 mm^2^ to 183.49 mm^2^, *b750-Rigid*: -125.35 mm^2^ to 139.65 mm^2^, *b750-Similarity*: -117.62 mm^2^ to 124.49 mm^2^), indicating that for 95% of cases the difference between the two methods' segmentation areas falls within this range. However, larger lesions were more prone to varying outside the limits of agreement, indicated by the dots outside the dotted lines in Fig. 6a). Reader-specific plots are in Supplemental Digital Content (Supplemental Fig. 3).

Mean segmentation distances remained low across methods (Fig. 6b) (*b50-Rigid*: 2.16 mm, *b750-Rigid*: 2.98 mm, *b750-Similarity*: 2.61 mm), though *b750-Rigid* had four outliers, one having a distance of above 20 mm. The Friedman test found no significant differences, indicating that co-registration did not substantially alter lesion position or size.

Inter-reader agreement (ICC3k) for segmentation area was high across methods: *no-coreg* (0.95), *b50-Rigid* (0.92), *b750-Rigid* (0.95), and *b750-Similarity* (0.93), with the highest consistency for *no-coreg* and *b750-Rigid*. For segmentation distances, agreement was lower, reaching only moderate to good levels (ICC3k: *no-coreg* = 0.63, *b50-Rigid* = 0.79, *b750-Rigid* = 0.73, *b750-Similarity* = 0.73), with the highest values obtained for the *b50-Rigid* method. Thus, distance-based agreement was notably lower than area-based agreement.

Pairwise comparisons of the segmentation distances using the Wilcoxon signed-rank test revealed significant differences only for reader 1 vs. reader 2 between *no-coreg* and *b750-Similarity* and reader 1 vs. reader 3 between *no-coreg* and *b750-Rigid*. Boxplots confirmed these findings, with no consistent pattern indicating a superior registration method (Fig. 6c). For area relations, the observations were similar. Significant differences in area relations were found only in two cases: between reader 1 and reader 2 comparing *no-coreg* and *b750-Rigid*, and between reader 1 and reader 3 comparing *no-coreg* and *b50-Rigid* (Fig. 6d).

#### Quantitative ADC values analysis

**Fig. 7:**
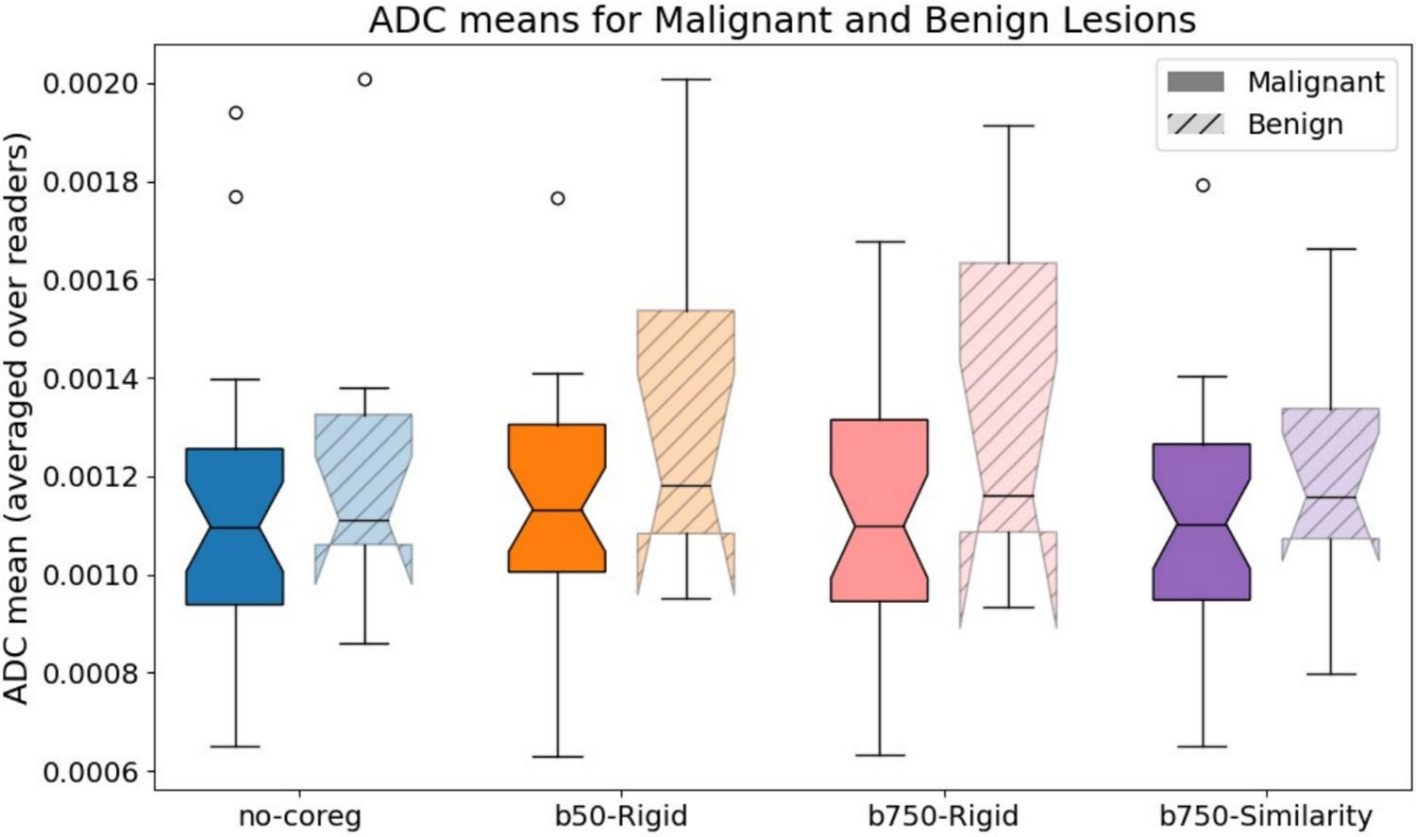
Boxplots for all methods (no-coreg and best performing co-registration methods) showing the ADC Mean for benign (hatched) and malignant (solid) lesions. No co-registration method is showing statistically significant differences from no-coreg

No statistically significant differences were found between the mean ADC values in the 2D lesion segmentation of *no-coreg* and the co-registration methods for benign and malignant lesions, as determined by repeated measures ANOVA with Dunnett's post hoc analysis (*p* > 0.05). Boxplots illustrating the distribution of ADC mean values for benign and malignant lesions across the different methods are shown in Fig. 7.

**Fig. 8:**
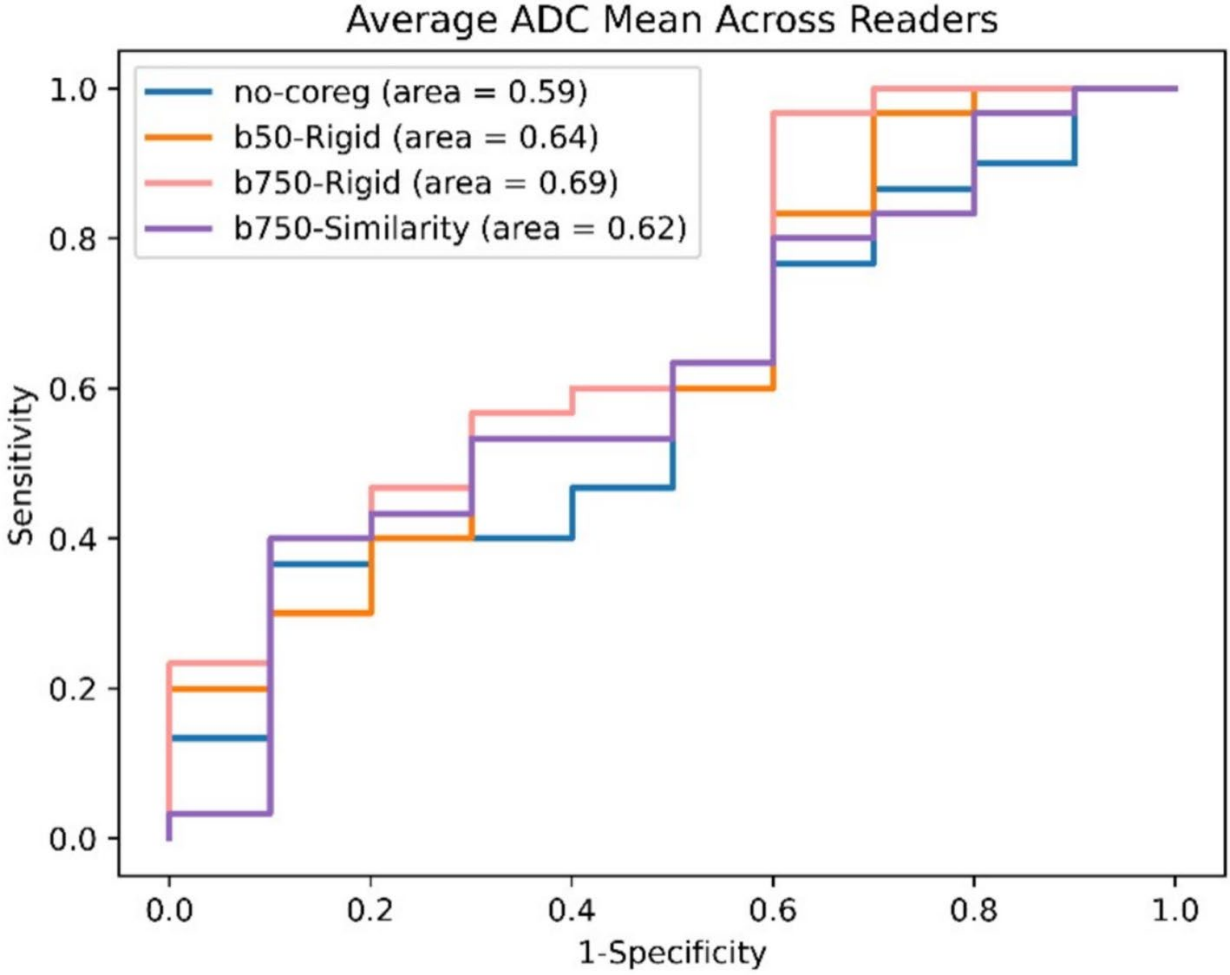
ROC curves based on ADC mean values averaged over the three readers for differentiating between benign and malignant breast lesions for the clinical analysis methods

We evaluated the diagnostic performance of the four segmentation methods using AUROC, sensitivity, specificity, and accuracy across three cutoff values. Figure 8 shows that *b750-Rigid* achieved the highest AUROC, with all co-registration methods outperforming *no-coreg*. Reader-specific ROC curves (Supplemental Fig. 4) confirmed this trend. Nevertheless, all AUROC values indicate discriminatory power below 0.7. To assess whether these differences were statistically significant, we performed pairwise comparisons of the AUROCs using DeLong’s test for correlated ROC curves. The comparisons between no-coreg and the other methods yielded p-values of 0.47 (*b50-Rigid*), 0.16 (*b750-Rigid*), and 0.69 (*b750-Similarity*). Although the numerical differences suggest a trend toward improved performance for all co-registration methods, these differences did not reach statistical significance, likely reflecting the limited sample size and the high correlation of predictions across methods.

Table 6 summarizes sensitivity, specificity, and accuracy for individual and A6702 trial thresholds. For the individual thresholds, the *b750-Rigid* method achieved the highest accuracy (82.5%) with a sensitivity of 96.7% and a specificity of 40%, outperforming all other methods. Notably, the *b50-Rigid* method showed comparable sensitivity (96.7%) but slightly lower accuracy (80%) due to reduced specificity. The *no-coreg* and *b750-Similarity* methods demonstrated higher specificity (90%) but at the cost of lower sensitivity (36.7% and 40% respectively) and accuracy (50% and 52.5% respectively).

**Table 6:** Sensitivity, specificity and accuracy for individual thresholds and A6702 trial thresholds for the clinical analysis methods

	Sensitivity	Specificity	Accuracy	Threshold (mm^2^/s)	McNemar p-value
**Individual thresholds**					
no-coreg	0.367	0.900	0.500	0.99 × 10^–3^	–
b50-Rigid	0.967	0.300	0.800	1.41 × 10^–3^	0.023
b750-Rigid	0.967	0.400	0.825	1.41 × 10^–3^	0.011
b750-Similarity	0.400	0.900	0.525	0.98 × 10^–3^	1.000
**Optimal thresholds according to A6702 trial**					
no-coreg	0.933	0.100	0.725	1.53 × 10^–3^	–
b50-Rigid	0.967	0.300	0.800	1.53 × 10^–3^	0.250
b750-Rigid	0.967	0.300	0.800	1.53 × 10^–3^	0.250
b750-Similarity	0.967	0.200	0.775	1.53 × 10^–3^	0.500
**Conservative thresholds according to A6702 trial**					
no-coreg	0.933	0.100	0.725	1.68 × 10^–3^	–
b50-Rigid	0.967	0.200	0.775	1.68 × 10^–3^	0.500
b750-Rigid	1.000	0.300	0.825	1.68 × 10^–3^	0.125
b750-Similarity	0.967	0.100	0.750	1.68 × 10^–3^	1.000

McNemar p-value indicating if there is statistical significance between the predictive accuracy of the co-registration methods and no-coreg at the different cutoffs

When applying the optimal thresholds according to the A6702 trial, all co-registration methods showed a high sensitivity of 96.7%, with *b750-Rigid* and *b50-Rigid* maintaining the highest accuracy (80%). The no-coreg approach had the lowest accuracy (72.5%) due to reduced specificity (10%) and lower sensitivity (93.3%). For the conservative thresholds, the *b750-Rigid* method achieved sensitivity of 100% and the highest accuracy with 82.5%.

Statistical significance in predictive accuracy according to McNemar’s test was demonstrated for the individually calculated optimal thresholds, showing the best individual accuracy, between *no-coreg* and *b50-Rigid* (*p* = 0.023) and *no-coreg* and *b750-Rigid* (*p* = 0.011).

## Discussion

This study evaluated the impact of different co-registration methods on the technical and diagnostic performance of breast MRI lesion assessment using diffusion-weighted imaging (DWI), focusing on whether appropriate registration techniques improve lesion differentiation while maintaining stable ADC values. Given that the misalignment we aim to reduce with these techniques can originate from multiple sources in DWI, including subject motion and distortion, we interpreted co-registration performance in the context of these combined effects.

Non-rigid registration methods (SyN) were excluded during the study due to instability of ADC mean values, possibly due to local distortions during the co-registration process. This problem was not visible in Affine, Rigid, and Similarity. In general, it should be noted that this study was conducted on a range of registration types, with the aim of gaining an understanding of the overall alignment power and its impact on lesion differentiation, rather than refining existing registrations to address the specific issue at hand. This also limited our ability to assess the effect of different similarity metrics as cost functions on the co-registration performance, as all transformations relied on the default mutual information cost function set by the ANTs library registration function.

The first part of the evaluation suggests that iterative registration of lower *b*-value images to b1500 may improve alignment and lesion differentiation compared to their non-iterative counterparts. Iterative methods generally showed lower p-values, fewer outliers, and reduced CoVs, indicating higher robustness. Unlike the non-iterative methods, they also tended to yield higher AUROC values, suggesting improved diagnostic performance alongside enhanced alignment. While the overall performance differences remain small, these findings suggest that iterative registration may enhance alignment due to its incremental approach, minimizing error-prone adjustments. Although research into these kinds of iterative registration strategies, in which each lower *b*-value image is incrementally aligned to the next, is limited, our findings are consistent with research demonstrating the benefits of iterative refinement within pairwise registration approaches. In classical image registration often an iterative optimization is used to gradually adapt the transformation parameters, thereby minimizing error by progressively adapting the alignment between a fixed and a moving image [35]. This is for example used in the Demons algorithm, which is an iterative non-rigid image registration method that gradually refines a dense deformation field, the Lucas–Kanade Algorithm, which iteratively refines a displacement field by estimating small motions, and even in the ANTs library itself iterative optimization of a similarity metric is the basis of registration [12, 36, 37]. These iterative schemes implicitly support the idea that gradual, controlled transformation updates improve spatial alignment. Our results on iterative registration suggest that the principle of iterative refinement during a pairwise registration algorithm can be applied beyond the scope of individual image pairs. Stepwise registration between similar images in contrast and image information, such as DWI images, enables small and more precise refinement at each stage, potentially limiting errors created by too big registration tasks and improving alignment to high *b*-value references [38].

A key open question is whether using high *b*-value DWI (b1500) as the registration target could enhance diagnostic differentiation. High *b*-value DWI has previously been suggested to support lesion detection in breast MRI [39, 40]. While b1500-based co-registration appeared to perform well, its advantage may stem from lesion segmentations being manually defined on b1500 DWI in the first analysis part of this study, potentially introducing bias. Due to the lack of external validation, this hypothesis remains unconfirmed.

Diagnostic performance analysis revealed that while initial sensitivity, specificity, and accuracy differences between the co-registration approaches were minimal without statistically significant difference, co-registration—especially *b750-Rigid*—showed improvements in a more clinically realistic setting with multiple readers and when including internal and external DWI breast MRI examinations. Therein the study included data from the ACRIN-6698 [23] dataset and a multi-reader multi-co-registration analysis. The performance disparity between datasets (*performance evaluation dataset* and *lesion segmentation dataset*) may stem from differences in segmentation approach, image quality, and dataset composition, since different DWIs with different signal-to-noise ratio (SNR) and quality can influence the ADC quality [41].

An additional observation was the variation in results compared to prior research published in WBIR [11]. Notably, even minor adjustments in the evaluation of the best methods—such as incorporating the binarization of the co-registered mask—had a significant impact on the analysis of the top-performing approaches.

A key finding was the consistently high sensitivity and low specificity across all methods when using A6702 trial thresholds. This is in line with previous studies investigating the A6702 thresholds, with a recent point-of-care analysis suggesting sensitivity of 92% and 95% for optimal and conservative thresholds, respectively and with a benign biopsy reduction rate of 18.6% and 12.4% [6]. This data was well matched in our analysis with 93.3% sensitivity and 10% specificity, which corresponds to the benign biopsy reduction, for the *no-coreg* method at both the optimal and conservative cutoffs. For the best performing method, *b750-Rigid*, we have a sensitivity of 96.7% and 100% (optimal and conservative cutoffs) and a specificity of 30% in both cutoffs. While a direct comparison to previous studies is not feasible due to differences in the datasets used, the comparable range of values suggests that there is no substantial mismatch in the performance of our approach.

It further needs to be considered that our study included a limited number of benign cases, which makes it difficult to draw strong conclusions. Despite promising results, particularly for *b750-Rigid*, further research is thus needed to determine the clinical applicability of co-registration. The primary objective of this feasibility study was to assess the potential value of further research in this area, and based on the results presented, further investigation appears justified. Furthermore, it would be worth investigating whether co-registration is particularly beneficial in cases with significant DWI misalignment, improving the differentiation between benign and malignant lesions.

Limitations of our study included the retrospective and rather limited clinical evaluation dataset, which, however, was thoroughly analyzed in a multi-reader evaluation addressing different co-registration approaches. Still larger studies are needed in order to validate the findings of our study, as the limited sample size is not able to reflect the diversity of lesion types and characteristics. Additionally, our in-house data only originated from a highly consistent MR acquisition protocol with minimal variations in the DWI sequence. However, these data were exclusively acquired on Siemens scanners, which reduces generalizability across vendors. To mitigate this, we restricted the external dataset to Siemens as well, ensuring comparability between datasets, while acknowledging that multi-vendor studies will be necessary to fully evaluate generalizability. The clinical reading only included the best performing co-registration techniques and not all co-registration sequences that were evaluated during the first phase of the study; thus it cannot be fully ruled out with regards to generalizability, there might be a different preferable co-registration technique. Further, our study only focused on DWI alone, not assessing the potential synergy of including additive MR sequences into the co-registration process (e.g., information from T2-weighted imaging). In diffusion-weighted imaging (DWI), artifacts arise not only from misalignments but also from factors such as intensity distortions, ghosting, and susceptibility effects [8, 10]. This study focused solely on misalignment-related artifacts, without accounting for other sources that may also affect ADC map accuracy and, consequently, diagnostic reliability in distinguishing benign and malignant lesions. Furthermore, ADC analyses alone still are not the only relevant factor for lesion characterization [42]. Another limitation of our study is the slight difference in *b*-values between datasets (750 vs. 800 s/mm^2^). Although prior studies suggest such differences have only limited influence on diagnostic performance of derived ADC maps, this variability should be acknowledged when interpreting our results [43].

In conclusion, the results indicate that co-registration, specifically *b750-Rigid*, can influence and potentially benefit lesion differentiation in breast MRI using ADC maps, with certain methods showing improvements in sensitivity, specificity, and accuracy when compared to ADC maps generated without prior alignment of *b*-value images. However, definitive recommendations for clinical implementation cannot yet be made, as performance variations were observed across datasets, and the improvements, while measurable, require validation in larger, prospective studies. Ultimately, this study supports the need for further research into optimized co-registration strategies for breast DWI MRI.

## Supplementary Information

Below is the link to the electronic supplementary material.

Supplementary file1 (DOCX 626 KB)

## Data Availability

Data generated or analyzed during the study are not available due to data protection regulations in the country.
