# Supplementary material for: Influence of co-registration on lesion characterization in diffusion-weighted breast MRI
Source: MAGMA. 2026 Jan 31;39(4):645–62. doi: 10.1007/s10334-026-01324-z (PMC13447411; doi:10.1007/s10334-026-01324-z)
Supplement: Supplementary file 1 [file 10334_2026_1324_MOESM1_ESM.docx]

Influence of co-registration on lesion characterization in diffusion-weighted breast MRI

Research Article in MAGMA

Luise Brock ^1,2,^*, M.Sc., Andrzej Liebert ^1,3^, Dr., Hannes Schreiter ^1^, M.Sc., Dominika Skwierawska ^1^, M.Sc., Chris Ehring ^1^, Jessica Eberle ^1^, Shirin Heidarikahkesh ^1^, M.Sc., Frederik B. Laun ^1^, Prof., Michael Uder ^1^, Prof., Lorenz A. Kapsner ^1,4^, MD, Judith Lach ^1^, Dr., Evelyn Wenkel ^5^, Prof, Sabine Ohlmeyer ^1^, MD, Dominique Hadler ^1^, MD, Florian Knoll ^2,6^, Prof., Sebastian Bickelhaupt ^1^, MD

^1^ Institute of Radiology, Universitätsklinikum Erlangen, Friedrich-Alexander-Universität Erlangen-Nürnberg (FAU), Erlangen, Germany

^2^ Department Artificial Intelligence in Biomedical Engineering, Friedrich-Alexander-Universität Erlangen-Nürnberg, Erlangen, Germany

^3^ Institute of Computer Science, Polish Academy of Science, Warsaw, Poland

^4^ Friedrich-Alexander-Universität Erlangen-Nürnberg, Medical Informatics, Erlangen, Germany

^5^ Diagnostische Radiologie, Radiologie München, München, Germany

^6^ Center for Advanced Imaging Innovation and Research (CAI2R), Department of Radiology, New York University Grossman School of Medicine, New York, New York, USA

* Corresponding author, e-mail: Luise.brock@uk-erlangen.de

**Supplemental Table 1** MR acquisition parameter for Diffusion-weighted images (DWI)

|  | **DWI** |
| --- | --- |
| Magnetic field strength | 3T; 1.5T |
| Slice thickness (mm) | 2.5 - 5 |
| Repetition time (ms) | 4100 - 9750 |
| Echo time (ms) | 54 - 106 |
| Inversion time (ms) | 0 - 250 |
| b-values (s/mm²) | 50, 750. 1500 |
| Matrix | 256×100-220×34-49 |
| Manufacturer | Siemens |

**Supplemental Fig. 1** Boxplots for all methods (*no-coreg* and co-registration methods) showing the ADC Mean for benign and malignant lesions. The red line indicates the median of the *no-coreg* boxplot and the methods marked orange are the ones differing statistically significantly from *no-coreg*

**Supplemental Chapter S1.** Masking Analysis for Registration

This supplementary section describes an additional analysis conducted to investigate whether restricting the co-registration cost function to the breast region improves registration performance and impacts the calculation of lesion ADC values on the performance evaluation dataset.

**S 1.1 Methods**

**S 1.1.1 Mask Generation**

Breast masks were generated automatically from the diffusion-weighted image (DWI) with the lowest b-value (b=50), as it provides the clearest anatomical representation among all DWI images. For each slice, a maximum-intensity projection (MIP) of adjacent slices was created to enhance breast tissue visibility. The resulting MIP was smoothed and binarized using a scaled Otsu threshold for an initial segmentation, which was subsequently refined using morphological opening and closing methods and Gaussian smoothing. The resulting mask covered the breast tissue while excluding background regions.

**S 1.1.2 Masked Co-registration Workflow**

The generated masks were incorporated into the co-registration process by restricting the calculation of the cost function to the masked region. All co-registration methods evaluated during performance evaluation were additionally performed with a mask-based cost-function and subsequently compared with the co-registration methods that didn’t use masking.

**S 1.1.3 Evaluation of Masked Co-registration**

To assess the impact of masking on lesion ADC mean values of the lesion segmentation, the ADC mean was calculated for each lesion after registration with and without masking. We evaluated whether masked registration changed lesion ADC means using paired t-tests, and whether it influenced diagnostic performance (sensitivity, specificity, accuracy) relative to the A6702 trial threshold using McNemar’s test.

**S 1.2 Results**

Several registration methods showed statistically significant differences in ADC mean when masking was applied. For benign lesions, significant changes were observed for b50-SyN, b1500-Affine, b1500-Rigid, b1500-Similarity, b1500-iter-Rigid, and b1500-iter-Similarity. For malignant lesions, significant changes were found for b750-SyN, b1500-Similarity, b1500-iter-Affine, b1500-iter-Similarity, and b1500-iter-SyN. These results are illustrated in Supplementary Figure 2, which displays boxplots comparing masked and unmasked ADC mean values.

The diagnostic performance, focusing on the accuracy, tended to decline when masking was applied (see Supplemental Table 2). In most co-registration methods, accuracy dropped when applying masking in the process, and in four of the sixteen cases, this reduction was statistically significant based on McNemar’s test. Only in a very small number of cases did masking lead to an improvement, and these occurred exclusively when SyN was used as the registration method. Notably, the b50-SyN co-registration method even produced a statistically significant gain in accuracy.

**S 1.3 Conclusion**

Based on these results, we conclude that masking the breast tissue for cost-function calculation does not improve co-registration in our dataset, except possibly for non-rigid registrations (SyN). While it is intuitive that masking might help at higher b-values due to increased noise, our additional analysis demonstrates that masking reduces diagnostic performance for rigid registrations, which are also the methods that performed better in the performance evaluation, and therefore does not provide a benefit in our study.

**Supplemental Figure 2.** Boxplots for all co-registration methods using once masks for calculating the co-registration metrics and once no masks are used, showing the ADC Mean for benign and malignant lesions. Statistical significance calculated using paired t-test between the lesion types unmasked and masked is indicated using * for p<0.05, ** for p<0.005 and *** for p<0.001.

**Supplemental Table 2.** Sensitivity, specificity and accuracy for the optimal threshold based on the A6702 trial threshold for the co-registration methods using once masks for calculating the co-registration metrics and once without masks. McNemar’s test is used for calculating statistical significance between the difference of masked and unmasked approaches in diagnostic accuracy.

|  | **Sensitivity** | **Specificity** | **Accuracy** | **McNemar‘s** |
| --- | --- | --- | --- | --- |
| **Optimal thresholds according to A6702 trial at 1.53x10-3 mm²/s** | | | | |
| b50-Affine | 0.942 | 0.320 | 0.681 | 0.039 |
| b50-Affine-mask | 0.870 | 0.280 | 0.626 |  |
| b50-Rigid | 0.957 | 0.380 | 0.714 | 0.031 |
| b50-Rigid-mask | 0.913 | 0.320 | 0.664 |  |
| b50-Similarity | 0.942 | 0.360 | 0.697 | 0.125 |
| b50-Similarity-mask | 0.913 | 0.300 | 0.655 |  |
| b50-SyN | 0.855 | 0.420 | 0.672 | 0.035 |
| b50-SyN | 0.913 | 0.52 | 0.748 |  |
| b750-Affine | 0.957 | 0.320 | 0.689 | 0.219 |
| b750-Affine-mask | 0.927 | 0.280 | 0.655 |  |
| b750-Rigid | 0.971 | 0.340 | 0.706 | 1.0 |
| b750-Rigid-mask | 0.957 | 0.340 | 0.697 |  |
| b750-Similarity | 0.971 | 0.320 | 0.697 | 0.125 |
| b750-Similarity-mask | 0.899 | 0.320 | 0.655 |  |
| b750-SyN | 0.884 | 0.280 | 0.630 | 0.146 |
| b750-SyN-mask | 0.971 | 0.280 | 0.681 |  |
| b1500-Affine | 0.986 | 0.280 | 0.689 | 0.125 |
| b1500-Affine-mask | 0.957 | 0.220 | 0.647 |  |
| b1500-Rigid | 0.971 | 0.340 | 0.706 | 0.012 |
| b1500-Rigid-mask | 0.928 | 0.220 | 0.630 |  |
| b1500-Similarity | 0.986 | 0.200 | 0.655 | 0.063 |
| b1500-Similarity-mask | 0.986 | 0.196 | 0.647 |  |
| b1500-SyN | 0.928 | 0.220 | 0.630 | 1.0 |
| b1500-SyN-mask | 0.913 | 0.240 | 0.630 |  |
| b1500-iter-Affine | 0.986 | 0.320 | 0.706 | 0.289 |
| b1500-iter-Affine-mask | 0.956 | 0.280 | 0.672 |  |
| b1500-iter-Rigid | 0.971 | 0.360 | 0.714 | 0.031 |
| b1500-iter-Rigid-mask | 0.942 | 0.280 | 0.664 |  |
| b1500-iter-Similarity | 0.971 | 0.340 | 0.706 | 0.635 |
| b1500-iter-Similarity-mask | 0.986 | 0.280 | 0.689 |  |
| b1500-iter-SyN | 0.899 | 0.340 | 0.664 | 1.0 |
| b1500-iter-SyNCC | 0.928 | 0.32 | 0.672 |  |

**Supplemental Table 3**  P- and t-values from ANOVA with Dunnett’s post hoc test for Coefficient of Variance (CoV) of ADC lesions separated in benign and malignant. Considering results with p-values < 0.05 as significant, marked bold

|  | CoV | | | |
| --- | --- | --- | --- | --- |
|  | **benign** | | **malignant** | |
|  | **t-value** | **p-value** | **t-value** | **p-value** |
| b50-Affine | 1.641 | 0.103 | 1.626 | 0.106 |
| b50-Rigid | 0.151 | 0.880 | -0.104 | 0.917 |
| b50-Similarity | 0.380 | 0.705 | 1.231 | 0.220 |
| b50-SyN | 3.323 | **0.001** | 3.176 | **0.002** |
| b750-Affine | 0.354 | 0.724 | -0.443 | 0.659 |
| b750-Rigid | -0.152 | 0.880 | -0.521 | 0.603 |
| b750-Similarity | -0.091 | 0.928 | -0.278 | 0.781 |
| b750-SyN | 2.409 | **0.017** | 2.261 | **0.025** |
| b1500-Affine | 2.021 | **0.045** | 2.855 | **0.005** |
| b1500-Rigid | 0.359 | 0.720 | 1.043 | 0.299 |
| b1500-Similarity | 0.535 | 0.594 | 1.353 | 0.178 |
| b1500-SyN | 3.579 | **4.82x10^-4^** | 4.237 | **3.96x10^-5^** |
| b1500-iter-Affine | 0.452 | 0.652 | 0.104 | 0.917 |
| b1500-iter-Rigid | -0.634 | 0.527 | -0.808 | 0.420 |
| b1500-iter-Similarity | -0.497 | 0.620 | -0.498 | 0.619 |
| b1500-iter-SyN | 1.796 | 0.075 | 2.193 | **0.030** |

**Supplemental Fig. 3** Agreement of lesion segmentations between no-coreg and individual co-registration methods (b50-Rigid, b750-Rigid and b750-Similarity) for the three readers. The dotted lines represent the limits of agreement (1.96 times the standard deviation above and below the mean) for each method and the dashed line represents the mean. Area in mm^2^

**Supplemental Fig. 4** ROC curves based on ADC mean values for differentiating between benign and malignant breast lesions. Each plot shows ROC curves for different methods to generate ADC maps, with and without co-registering the individual b-values before ADC map generation
